# Magnetoencephalography for the Detection of Intervention Effects of a Specific Nutrient Combination in Patients with Mild Alzheimer’s Disease: Results from an Exploratory Double-Blind, Randomized, Controlled Study

**DOI:** 10.3389/fneur.2016.00161

**Published:** 2016-10-17

**Authors:** Elisabeth C. W. van Straaten, Hanneke de Waal, Marieke M. Lansbergen, Philip Scheltens, Fernando Maestu, Rafal Nowak, Arjan Hillebrand, Cornelis J. Stam

**Affiliations:** ^1^Department of Clinical Neurophysiology, MEG Center, VU Medical Center, Amsterdam, Netherlands; ^2^Nutricia Advanced Medical Nutrition, Nutricia Research, Utrecht, Netherlands; ^3^Department of Neurology, Alzheimer Center, VU Medical Center, Amsterdam, Netherlands; ^4^Laboratory of Cognitive and Computational Neuroscience (UCM-UPM), Center for Biomedical Technology, Madrid, Spain; ^5^Magnetoencephalography Unit, Centro Medico Teknon, Barcelona, Spain

**Keywords:** magnetoencephalography, brain networks, clinical trial, Alzheimer’s disease, medical nutrition

## Abstract

Synaptic loss is an early pathological finding in Alzheimer’s disease (AD) and correlates with memory impairment. Changes in macroscopic brain activity measured with electro- and magnetoencephalography (EEG and MEG) in AD indicate synaptic changes and may therefore serve as markers of intervention effects in clinical trials. EEG peak frequency and functional networks have shown, in addition to improved memory performance, to be sensitive to detect an intervention effect in mild AD patients of the medical food Souvenaid containing the specific nutrient combination Fortasyn^®^ Connect, which is designed to enhance synapse formation and function. Here, we explore the value of MEG, with higher spatial resolution than EEG, in identifying intervention effects of the nutrient combination by comparing MEG spectral measures, functional connectivity, and networks between an intervention and a control group. Quantitative markers describing spectral properties, functional connectivity, and graph theoretical aspects of MEG from the exploratory 24-week, double-blind, randomized, controlled Souvenir II MEG sub-study (NTR1975, http://www.trialregister.nl) in drug naïve patients with mild AD were compared between a test group (*n* = 27), receiving Souvenaid, and a control group (*n* = 28), receiving an isocaloric control product. The groups were unbalanced at screening with respect to Mini-Mental State Examination. Peak frequencies of MEG were compared with EEG peak frequencies, recorded in the same patients at similar time points, were compared with respect to sensitivity to intervention effects. No consistent statistically significant intervention effects were detected. In addition, we found no difference in sensitivity between MEG and EEG peak frequency. This exploratory study could not unequivocally establish the value of MEG in detecting interventional effects on brain activity, possibly due to small sample size and unbalanced study groups. We found no indication that the difference could be attributed to a lack of sensitivity of MEG compared with EEG. MEG in randomized controlled trials is feasible but its value to disclose intervention effects of Souvenaid in mild AD patients needs to be studied further.

## Introduction

Alzheimer’s disease (AD) is the leading cause of dementia, and although it has an incompletely understood etiology, synaptic connectivity seems to be reduced already in early stages (1, 2). Synapses are the main structures for functional connectivity between neurons, and loss of this connectivity is related to impaired cognition in AD (3). Therefore, optimizing synapse formation and function may counteract some of the cognitive effects of the AD process.

Synapses consist of a large part of neuronal membrane, and formation and function of neuronal membrane can be enhanced by increasing the availability of specific nutrients that are required for the synthesis of phospholipids [for an overview, see Ref. (4)]. In many *in vitro* and *in vivo* studies, the combined administration of these nutrients has been shown to increase brain levels of phospholipids and synaptic proteins, to increase synaptic density, to enhance cholinergic neurotransmission and receptor functioning, and to improve functional brain connectivity and cognitive performance [e.g., Ref. (5–11)].

The medical food Souvenaid contains the specific nutrient combination Fortasyn Connect which was designed to counteract synapse loss and dysfunction in AD by supplying precursors and cofactors that are essential for neuronal membrane formation and maintenance and that are believed to be insufficiently available in AD, i.e., docosahexaenoic acid (DHA), eicosapentaenoic acid (EPA), uridine (in the form of uridine monophosphate), choline, phospholipids, folic acid, vitamins B6, B12, C, E, and selenium.

Souvenaid is intended as a medical food for oral consumption under medical supervision with the purpose of addressing disease-specific nutrient requirements. It has been found to positively affect memory function in mild AD (12–14).

Neurophysiological measures aid in the interpretation of the mode of action of an intervention (15). A previous clinical study showed preserved EEG spectral and graph theory-based functional network measures in patients receiving Souvenaid compared with a control group in a double-blind, randomized, controlled trial, indicating preserved connectivity as a macroscopically measurable objective intervention effect (14). Recent research suggests that the AD effect is not homogeneously distributed over different brain regions and that, in terms of brain networks, especially highly connected hub regions are affected (16–18). More spatially detailed information in these hub areas could therefore potentially disclose additional information on the mode of action of the intervention. Magnetoencephalography (MEG) combines a high temporal resolution with a relatively high spatial resolution. MEG signals are hardly disturbed by the skull, and absolute values can be used without the need for a reference. This allows the study of brain activity in great spatial detail and a reliable transformation to anatomical space.

Magnetoencephalography recordings in AD patients have mainly shown loss of functional connectivity of fast (>8 Hz) oscillatory brain activity and a less optimal network structure (18–22). However, no intervention studies have been performed in AD patients to improve the abnormalities seen with MEG recordings. We therefore set out to examine in an exploratory setting the feasibility and value of MEG to detect intervention effects of a medical food on brain activity in mild AD patients.

## Materials and Methods

### Study Design and Subjects

Fifty-five patients aged ≥50 years, meeting the criteria for AD according to the National Institute of Neurological and Communicative Disorders and Stroke and the Alzheimer’s Disease and Related Disorders Association (NINCDS-ADRDA) criteria and with a Mini-Mental State Examination (MMSE) ≥20 were enrolled in the Souvenir II MEG sub-study, which had an exploratory, randomized, controlled, double-blind, parallel-group design. Participants were 1:1 randomly allocated to either the test (Souvenaid, containing Fortasyn Connect) or the isocaloric control product (without Fortasyn Connect) as a 125-ml daily drink for 24 weeks based on a computer-generated randomization list. Patient inclusion and exclusion criteria were identical to those previously described in the Souvenir II study (14). Assessments were done at baseline, week 12, and week 24, including MEG, EEG, blood sampling, safety, and compliance based on diaries. Participants were recruited from one center in Amsterdam (*n* = 47), one center in Madrid (*n* = 2), and one center in Barcelona (*n* = 6). The study was conducted in accordance with the Declaration of Helsinki, the International Conference on Harmonization (ICH) guidelines for Good Clinical Practice (GCP) as appropriate for nutritional products, and local legislation of the country in which the research was conducted. The Dutch Trial Registration number for this study is NTR1975. The ethical review boards of the local study sites approved the study. Written informed consent was obtained from patients and caregivers.

### MEG Recording and Post-Processing

**Figure 1:**
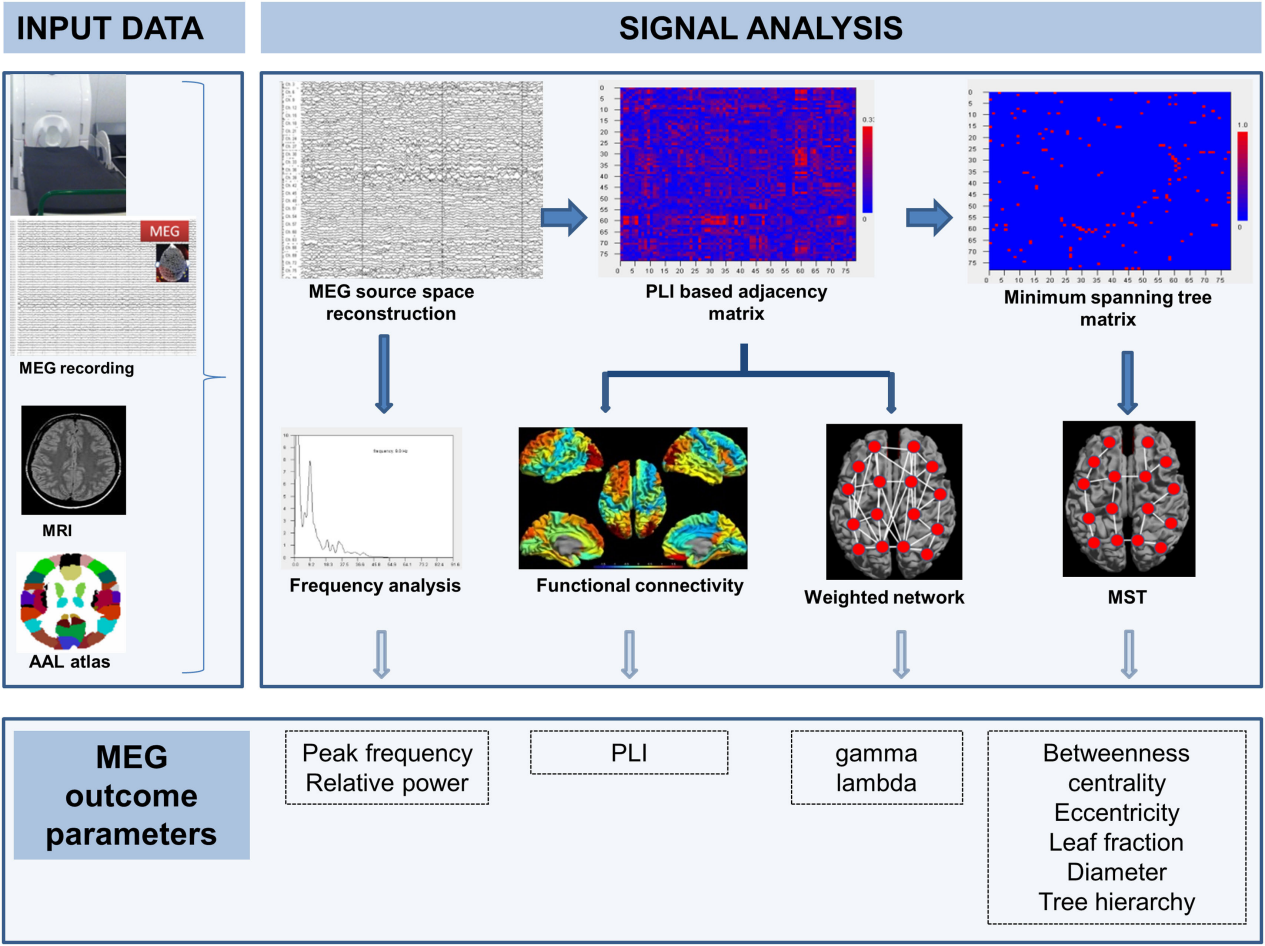
**MEG recording and analysis pipeline**. Arrows indicate steps in time. Source-space MEG of the patient was reconstructed using the signal-space MEG time series, the patient’s MRI, and an anatomical atlas (input data). MEG was used for frequency analysis (with outcome parameters: peak frequency and relative power) and the construction of a functional connectivity-based (PLI) adjacency matrix. From this matrix, mean PLI as well as weighted network measures gamma and lambda were computed and the minimum spanning tree (MST) matrix was derived. From the MST matrix, network metrics betweenness centrality, eccentricity, leaf fraction, diameter, and tree hierarchy were computed. All outcome measures were compared between groups.

Figure 1 shows an overview of the MEG recording and analysis steps. Twenty-minute eyes-closed resting-state MEG data were recorded using whole head MEG systems inside a magnetically shielded room in three centers (Table 1). The head position within the scanner was determined by using four attached head position indicator coils. The positions of the indicator coils and the head shape were determined before the recordings using a digitizer (3Space Fast-Track, Polhemus, Colchester, VT, USA). The recorded MEG data were transferred to the Amsterdam center for central post-processing. Channels with poor signal quality (maximum of 10) were manually de-selected after visual inspection for artifacts by a trained technician, and additionally a spatio-temporal Signal-Space Separation (tSSS) filter (Elekta data) was applied for artifact removal (10 s window, activity–noise correlation set at 0.9) using MaxFilter software (Elekta-Neuromag Oy version 2.2.10) (23). Head movement and number of discarded channels did not differ between recording centers.

**Table 1:** **Descriptive data of the recording MEG laboratories**.

	Department of Clinical Neurophysiology, MEG Center, VU Medical Center	Laboratory of Cognitive and Computational Neuroscience (UCM-UPM), Center for Biomedical Technology	Magnetoencephalography Unit, Centro Medico Teknon
City, country	Amsterdam, the Netherlands	Madrid, Spain	Barcelona, Spain
MEG system	Elekta	Elekta	4D Neuroimaging
Number of subjects	47	2	6
Sensor types	102 magnetometers and 204 planar gradiometers	102 magnetometers and 204 planar gradiometers	146 magnetometers
Sampling rate (Hz)	1250	1000	678
Online filtering (pass band, Hz)	0.1–410	0.03–330	1–70

A beamformer method was used to transfer the signal-space data of the Elekta MEGs (*n* = 49 subjects) to source-space for further analysis: the MEG signals, recorded at the sensors, were reconstructed to MEG signals originating from 78 sources [according to the 78 automated anatomical labeling (AAL) areas] (24–26). Therefore, magnetic resonance imaging (MRI) T1 series of the patients were coregistered on the MEG data using the current head position and digitized scalp, as well as the outline of the scalp as obtained from the MRI outline data. For nine subjects, no suitable MRI was available. For these subjects, the best fitting MRI was identified out of the MRIs available from this study and used for matching with satisfying matching results. The MEGs recorded using 4D Neuroimaging (Barcelona, *n* = 6) were analyzed separately in the original signal-space, due to incompatible software requirements for source-space computation. Five artifact-free epochs of 4096 samples (down-sampled four times, 13.1–16.4 s) were selected from each recording by a trained MEG technician and analyzed with in-house developed open access software (BrainWave, version 0.9.72, C. J. Stam, http://home.kpn.nl/stam7883/brainwave.html).

For the source-space MEGs, outcome measures included measures for analysis A (characterized by one average value per subject per time point, see Statistical Analysis) and measures for analysis B (with one value for each AAL region per MEG study, see Statistical Analysis). The measures for analysis A included mean occipital peak frequency (for comparison with the previous Souvenir II EEG study and defined as the median frequency between 4 and 13 Hz, averaged across the signals of the occipital regions), relative power in the different frequency bands (averaged over all brain regions), functional connectivity [assessed with the Phase Lag Index (PLI) and averaged over all brain regions], and several functional network measures [mean normalized clustering coefficient (gamma), the averaged normalized shortest path length (lambda), and minimum spanning tree (MST) network-derived measures (leaf fraction, diameter, tree hierarchy, maximum betweenness centrality, and maximum eccentricity)]. The measures for analysis B included non-averaged region-specific peak frequency, relative power, functional connectivity, and MST betweenness centrality and eccentricity (27–30). Analyses were performed in six frequency bands (delta 0.5–4 Hz, theta 4–8 Hz, alpha1 8–10 Hz, alpha2 10–13 Hz, beta 13–30 Hz, and gamma 30–48 Hz). Functional connectivity quantifies the functional interactions between brain regions with high values indicating a strong functional connection and low values indicating a weak or absent functional connection. The MSTs were constructed in such a way that all network nodes were included in the graph using the connections with the highest functional connectivity and without the formation of loops (29). Only descriptive statistics with respect to relative power and peak frequency were analyzed for the signal-space MEG subsample (Barcelona).

### *Post Hoc* Analysis

In addition to MEG, the participants underwent 20-min eyes-closed task-free EEG according to the protocol used in the previous Souvenaid II study just before or after the MEG measurement (14). In short, digital EEG was recorded according to the 10–20 system with 21 electrodes and sample frequency 500 Hz (BrainRT, OSG Belgium). To compare source-space MEG’s sensitivity in detecting an intervention effect to that of EEG, we additionally analyzed peak frequency based on sensor-level EEG data from the same study sample (*n* = 49) using a common or average reference, online filter settings high pass 0.16 Hz and low pass 70 Hz. As for MEG, EEG peak frequency was computed for the parieto-occipital electrodes (P3, P4, O1, and O2) as the median frequency between 4 and 13 Hz. Only peak frequency was assessed since this parameter had the most clearly interpretable significant results in the previous Souvenir II EEG study and is (contrary to functional connectivity and network measures) not dependent on number of electrodes/sensors.

### Safety and Compliance

Safety assessments included the examination of patient medical history, recording of (serious) adverse events [(S)AE], and the monitoring of vital signs and additional laboratory parameters (liver panel and renal function). Study product compliance was assessed using a daily diary, which was completed by the subject or caregiver at home.

Magnetoencephalography analyses were done on the intent-to-treat (ITT) MEG population (*n* = 49), defined as all randomized patients (ITT) with source-space reconstructed MEGs. Safety analyses were performed on the all-subjects treated (AST) population (*n* = 55), defined as all randomized patients who received at least one dose of study product.

### Statistical Analysis

No previous study results were available on intervention in AD patients to improve abnormalities seen with MEG recordings on which the sample size calculation could be based. With the proposed sample size of 20 patients per group, a treatment difference of about 1 SD can be detected with a power of 90% using *t*-test and a two-tailed alpha of 0.05.

Source-space MEG outcome parameters that yield one value per subject per time point (Analysis A: mean occipital peak frequency, mean PLI, *gamma, lambda*, leaf fraction, diameter, tree hierarchy, maximum betweenness centrality, and maximum eccentricity) were analyzed using mixed models for repeated measures (MMRM) with change-from-baseline as an outcome, using SAS^®^ software (SAS Enterprise Guide 4.3 for Windows, SAS Institute Inc., Cary, NC, USA) and in line with the previous Souvenir II EEG sub-study. More specifically, a mixed model by specifying the within subject unstructured variance–covariance structure and including group (treatment arm), time (as continuous variable; treatment duration), and the group × time interaction as fixed effects, adjusted for baseline values (baseline was included as covariate) was used. “Site” was not included as random effect, because MEG data for the primary MEG analysis (based on “source-space”) were only available from two sites and the majority of the data were collected at one single site. Normality of the distribution was checked, and no transformations to correct for lack of normality were needed. The two degrees of freedom contrast (for group and group × time) describing the difference in trajectories over time between Souvenaid and control groups was taken as the primary indication of treatment effect during the intervention period.

Source-space MEG outcome parameters that yield one value per AAL region per subject per time point (analysis B: peak frequency, relative power, PLI, MST betweenness centrality, and MST eccentricity) were analyzed with two different approaches: (1) to adhere to the analysis method of the previous Souvenir II EEG sub-study and to take into account covariates and evolution over time, a statistical analysis with average values for 10 predefined brain areas [consisting of 2 (left and right) times 5 (frontal, central, temporal, parietal, and occipital) regions] to examine whether an interaction of treatment and time was constant over the 10 brain areas with brain area as additional fixed factor and (2) to use the higher spatial resolution of the MEG, a permutation analysis with each of the 78 AAL regions, correcting for multiple comparisons to indicate the brain regions responsible for group differences. With this method, we derived a null distribution for between-group differences by permuting group assignment and calculating a *t*-statistic after each permutation with an independent *t*-test (31). The maximum *t*-value across ROIs of each permutation was used to construct a distribution of maximum *t*-values of 1000 permutations. To correct for multiple comparisons, we used the single threshold test with *p*-value set at 0.05.

## Results

### Subject Flow

**Figure 2:**
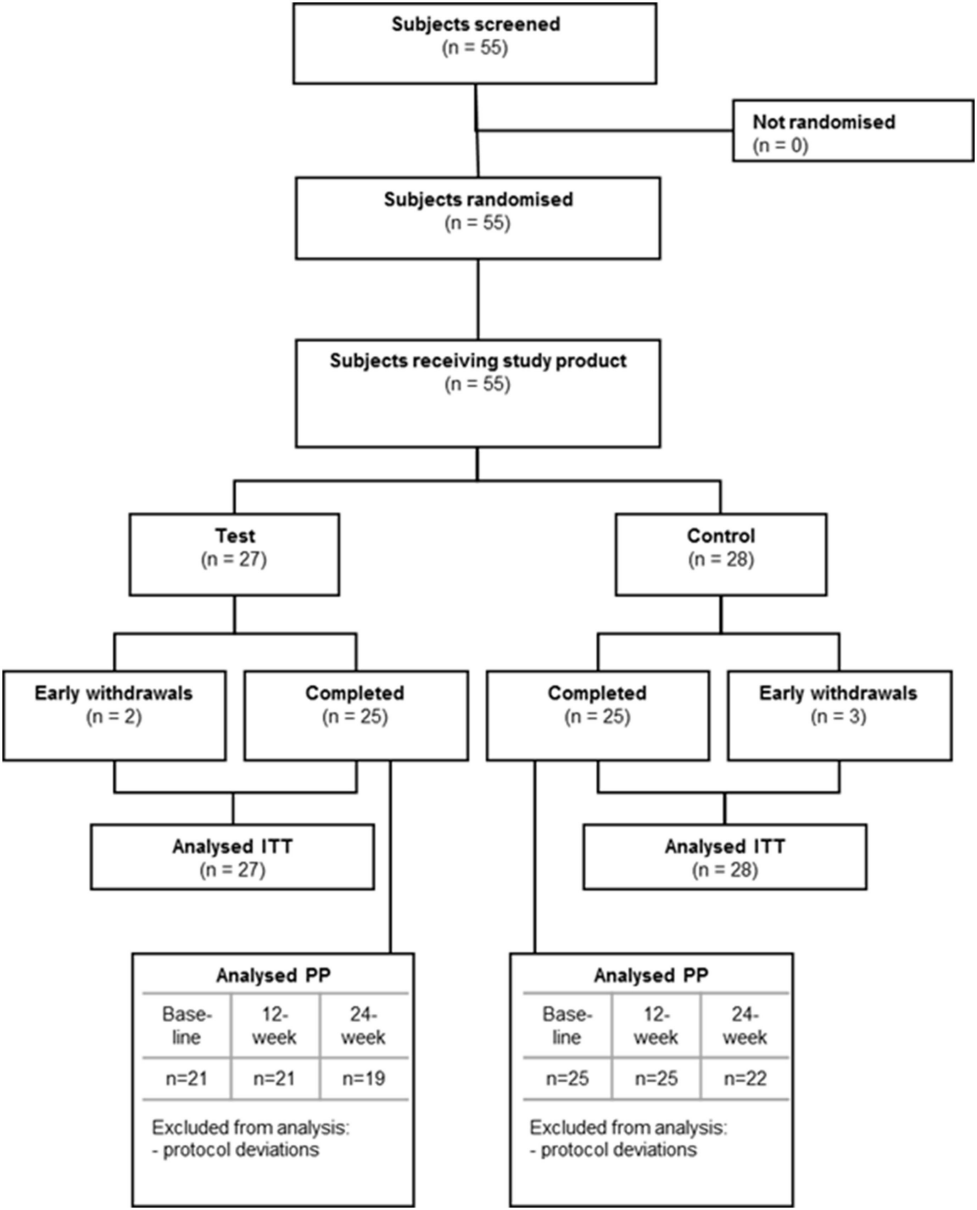
**Subject flow chart**. *MEG analysis population, excluding six subjects from Barcelona.

Figure 2 and Table 2 show the flow and demographic data of subjects throughout the study for both study groups, separately for the total ITT population and the ITT MEG analysis population. Five subjects (9%) withdrew from the study early (*n* = 2 subjects from the test group and *n* = 3 subjects from the control group). Reasons for early withdrawal were withdrawal of consent (*n* = 2, 1 in each group), occurrence of SAEs (*n* = 2, 1 in each group), and lost to follow-up (*n* = 1 in the control group). Subjects were well balanced over the study groups with respect to baseline characteristics, except for the total MMSE score. Despite the randomization procedure, mean total MMSE score at screening was significantly higher for the control group compared with the test group in both ITT populations (total ITT population: *t*-test, *p* = 0.021; ITT MEG analysis population: *t*-test, *p* = 0.032), indicating worse cognition at screening in the test group as compared with the control group.

**Table 2:** **Baseline demographics and characteristics of the intent-to-treat (ITT) populations**.

	Total ITT population		ITT MEG analysis population	
	Control *n* = 28	Test *n* = 27	Control *n* = 27	Test *n* = 22
Age, years [range]	68.4 (7.9) [56–85]	69.7 (7.9) [53–87]	68.1 (7.9) [56–85]	68.4 (7.6) [53–80]
Male, *n* (%)	14 (50.0%)	15 (55.6%)	14 (51.9%)	12 (54.5%)
Years of education beyond primary school	6.2 (3.9)	7.6 (4.4)	6.2 (4.0)	7.2 (4.2)
Duration AD since diagnosis, months, median [range]	2.0 [0.0–46.0]	2.0 [0.0–29.0]	2.0 [0.0–46.0]	3.0 [0.0–29.0]
Total MMSE score	25.1 (2.8)	23.6 (2.1)	25.3 (2.6)	23.8 (2.3)

*Data are presented as mean (SD) unless stated otherwise*.

*AD, Alzheimer’s disease; MMSE, Mini-Mental State Examination*.

### MEG Results

**Figure 3:**
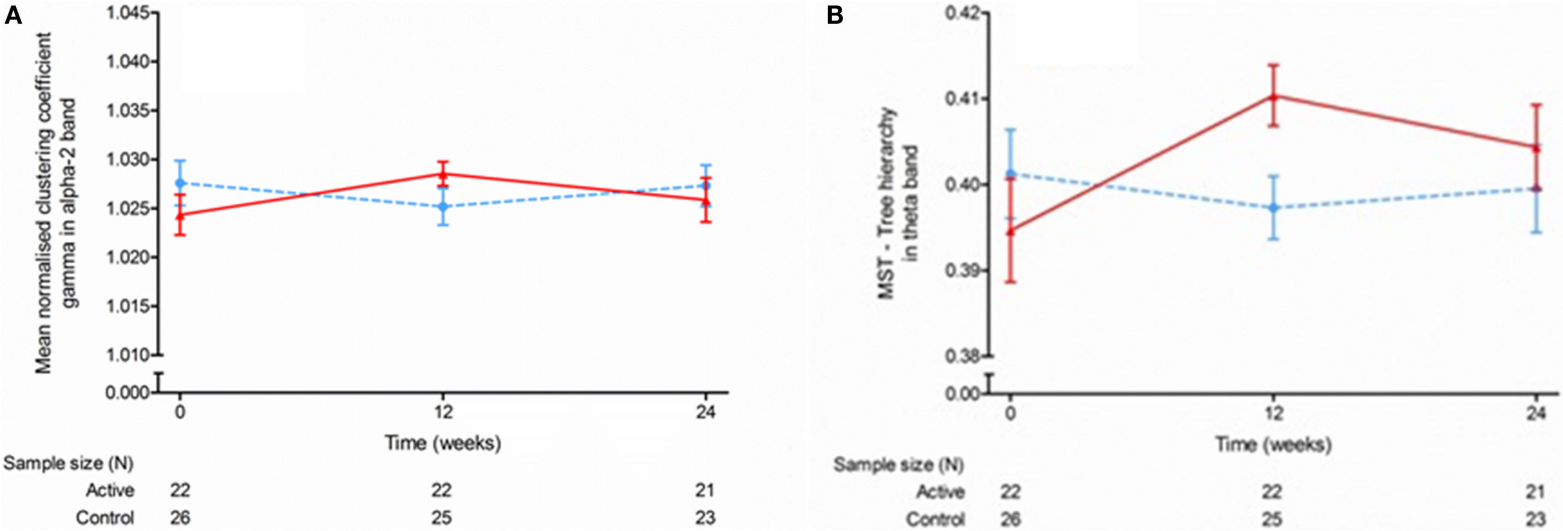
**Mean normalized clustering coefficient (gamma) in the alpha 2 band (A) and minimum spanning tree hierarchy in the theta band (B) at baseline, week 12, and week 24**. Data are raw means ± SEM.

Peak frequency and relative power of the source-space data were not different between the groups over the 24-week intervention period with any of the statistical methods (Appendices A and B in Supplementary Material). Mean functional connectivity as measured with PLI was not different between groups (MMRM, Appendix C in Supplementary Material), but with regional analysis using permutation testing, the test group showed significant lower theta band values in the left gyrus rectus (permutation test; *p* < 0.05). The network measures were generally not different between the groups except that the MMRM showed a group difference over time for normalized clustering coefficient gamma in the alpha-2 band [MMRM; *F*(2,43) = 3.91, *p* = 0.028] and tree hierarchy in the theta band [MMRM; *F*(2,43) = 5.38, *p* = 0.008], but at endpoint no differences were observed between study groups (Figure 3; Appendices D–J in Supplementary Material). In addition, the permutation analysis showed a significantly lower theta band MST betweenness centrality at baseline in the left gyrus rectus in the test group compared with the control group (permutation test; *p* < 0.05). Descriptive statistics for mean peak frequency and relative power of the MEGs in signal-space (from Barcelona, *n* = 6) are presented in Appendices K and L in Supplementary Material.

### *Post Hoc* Results

In agreement with the MEG results, EEG peak frequency analysis yielded no group differences, suggesting that MEG is not necessarily less sensitive than EEG to detect an intervention effect.

*Post hoc* analyses were conducted to test the influence of gender on the intervention effect on the MEG outcome parameters. We could not demonstrate an intervention effect in males or females when tested separately.

As significant differences were observed in screening MMSE between the study groups, additional statistical analyses were conducted for all MEG parameters adjusting for screening MMSE. The results from these confounder analyses were similar to the results from the primary MEG analyses, indicating no straightforward confounding effects of screening MMSE.

### Safety and Compliance

Regarding safety, no clinically relevant differences between groups were found for the blood safety parameters, (S)AE occurrence, and vital signs assessments. The calculated subject reported compliance during 24 weeks was high with 91% in the test group and 98% in the control group (Student’s *t*-test, *p* = 0.19).

## Discussion

### Summary

This Souvenir II MEG sub-study was a 24-week study in 55 patients with mild AD to explore whether MEG has the potential to detect effects resulting from 24 weeks’ intake of Souvenaid compared with a control product. Based on results from a previous EEG study, we expected group differences in the peak frequency, delta band functional connectivity, and beta band network parameters gamma and lambda (14, 32). However, we observed no endpoint differences in the MEG measures between the test group and the control group. Although some differences in trajectory over time were found (gamma in the alpha2 band and MST tree hierarchy in the theta band), these findings were not confirmed by differences at endpoint and did not reveal a consistent pattern that was anticipated based on the previous EEG results, existing knowledge on AD pathology, and the hypothesized mode of action of Souvenaid. Due to the number of tests, we anticipate that these results may have resulted from type 1 errors. The study product was safe and well tolerated, as has been shown previously in mild and in mild-to-moderate AD (13, 14).

### Relationship between Souvenaid, Synapses, and Electrophysiological Changes in AD

The main mechanism for neuronal communication is synaptic transmission of electrical current. Synapses as specialized parts of the cell membrane are built to facilitate transmission of current by continuous excitatory and inhibitory input from sending axons to receiving dendrites. As a result, postsynaptic potentials are constantly changing, and the net fluctuation of the electrical field can be measured as oscillations of the magnetic field with MEG and of the electrical field with EEG. The most consistent electrophysiological finding in AD is slowing of the oscillatory activity (33). The exact mechanism of this phenomenon is unknown, but model studies indicate that loss of synaptic connectivity plays a role (18, 34). In addition, breakdown of functional network integrity has also been found in EEG (35, 36) and could be modeled by decreasing coupling between neuronal assemblies in a computer model (37). The effects of the Souvenaid on brain function in early AD have been assessed before with EEG and preserved peak frequency and network organization were related to the nutritional intervention (14, 32). Given the effects of the Fortasyn Connect nutrients on synaptic structure and function and on brain connectivity in animal studies (5, 6, 10, 11) and the known changes on EEG in AD patients, it was hypothesized that improved synaptic function due to the intervention may be picked up by EEG as preserved oscillatory frequency and preserved functional network organization. In essence, MEG and EEG both measure the macroscopic effects of postsynaptic potential changes. It was, therefore, expected that the same neurophysiological principles and intervention effects apply to MEG.

### Control Group Results

Our control group did not behave as expected. From previous neurophysiological studies, it is clear that AD is related to noticeable macroscopic neurophysiological changes: the peak frequency and functional connectivity decrease over time, and network parameters indicate a more random organization of functional connections (15). In our control group, we could not observe most of these changes during the course of the study, indicating that our control group was not typical during the course of the study. On the other hand, 24 weeks may be too short to detect neurophysiological decline in a relative small group of AD subjects and therefore longer study duration may reveal the typical AD-related MEG changes.

### MEG in Relation to EEG

Although the choice of the imaging technique has a known influence on the results (38), we did not find indications of lower sensitivity of MEG compared with EEG. EEG was part of the study protocol, and in a *post hoc* analysis, we analyzed EEG peak frequency from the same study population at the same time points as the MEGs using the same software and comparable (i.e., occipital) cortical areas. Peak frequency is one of the most robust and most investigated neurophysiological measures in AD and behaves in a similar way in EEG and MEG with lower values in AD compared with controls (15, 20, 39–41). In addition, EEG peak frequency has previously been identified as a sensitive measure for group differences after 24 weeks of intervention with Souvenaid (14). However, in the current study, EEG peak frequency did not differ between the test and control groups over the course of the study and at endpoint. Therefore, we could not attribute the lack of group difference to a lower sensitivity of MEG compared with EEG. On the other hand, MEG, which is more costly, more technical demanding, and less widely available than EEG, did not perform superior to EEG in this study. MEG may theoretically outperform EEG given several technical differences: magnetic fields detected with MEG do not suffer from filtering effects of surrounding tissue as much as the electric fields detected with EEG. Therefore, the estimation of sources, especially when located in deeper brain structures is more reliable and the maximum spatial and temporal resolution is higher. Another potential advantage of MEG over EEG is the fact that no referential recording point is needed. The use of a reference has been shown to influence the results, especially in EEG functional connectivity and network studies. Despite these advantages, we could not establish a higher sensitivity for an intervention effect over 24 weeks.

### Limitations

Several methodological limitations can be identified. The data from different centers were not compatible when using the latest analysis techniques. The field of MEG analysis is rapidly developing, and the demands on the recordings and the analysis strategies change accordingly. At this moment, no uniform MEG data format is available, and recordings from different scanner types cannot be pre-processed similarly. Not all state-of-the-art techniques are available and validated for merging data from different MEG systems. In the current study, we decided to implement the latest analysis technique for Elekta MEGs at the cost of excluding several subjects from the analyses. This has led to a reduction of power, but we feel that the accuracy of the source-space analysis method compensated for this loss (26). However, next steps toward a uniform MEG data format will likely increase the applicability of MEG in clinical trials.

In the current study, we did not control for time of the recording. Brain oscillations as measured with EEG and MEG are known to vary over the course of 24 h, with the lowest power of the signal early in the morning (42–44). However, this diurnal rhythm applies to absolute oscillatory power and to a large extend is similar for all frequency bands. In our study, we only used relative frequency measures for the spectral analyses. It can, therefore, be expected that circadian effects will largely cancel out in these types of analyses, and we feel that the present results are not influenced substantially by differences in time of recording.

This study was exploratory in nature with small subject groups, which has some known drawbacks when interpreting group differences, some of them being the larger risk of non-uniformity of the groups and the lack of statistical power. Unfortunately, the patient groups were found to be unbalanced with respect to screening MMSE, despite a careful computerized randomization process. Covariate analyses correcting for the baseline differences did not reveal confounding effects. However, these adjustments assumed a linear relationship between the baseline characteristic and the outcome, whereas in reality, the relationship between MMSE at screening and the outcome parameters might have been more complex. We controlled for some patient characteristics that have a well-known effect on brain oscillations, such as use of benzodiazepines and structural brain lesions, including infarctions and tumors. However, other, unknown, factors, such as brain atrophy or small vessel disease, might have biased the results. It, therefore, remains unclear to what extent the intervention effect was masked by group differences already present at screening.

## Conclusion

Possibly due to methodological issues (unbalanced and small study groups), we were not able to assess the potential of MEG-based measures as markers of an intervention effect of a medical food in mild AD patients. Furthermore, we could not replicate the EEG results from the well-powered Souvenir II study (*n* = 259) in which a significant effect of Souvenaid compared with control product was shown on peak frequency, functional connectivity, and brain network organization in patients with mild AD (14, 32). We did not demonstrate consistent statistically significant differences between groups for additional regional-specific MEG outcome measures nor did we observe the typical AD deterioration over time in the control group. Substantially increasing the sample size for better-balanced study groups might give indications in this direction.

Magnetoencephalography did not seem less sensitive than EEG but with a greater ability of in-depth regional analysis. The potential of MEG analysis might be demonstrated in future, larger, clinical studies when the field has progressed toward a more unified data format. Moreover, newly developed MEG analyses with hippocampal and centroid voxel MEG virtual electrodes as well as other (directed) connectivity and network analysis can be applied to further characterize brain magnetic activity in AD.

## Author Contributions

ES was involved in the design of the study, writing of the protocol, acquisition, analysis, and interpretation of the data, and wrote the protocol. HW contributed to the acquisition, analysis, and interpretation of the data. ML participated in design of the study, writing of the protocol, analysis, and interpretation of the data. PS and CS made contributions to the design of the work and interpretation of the data. FM, RN, and AH were involved in the design, acquisition, and interpretation of the data. All authors contributed to and have approved the final manuscript.

## Conflict of Interest Statement

ML is an employee of the funding body. PS has received grant support (for the institution) from GE Healthcare, Danone Research, Piramal, and MERCK. In the past 2 years, he has received consultancy/speaker fees (paid to the institution) from Lilly, GE Healthcare, Novartis, Forum, Sanofi, Nutricia, Probiodrug, and EIP Pharma. The remaining coauthors declare that the research was conducted in the absence of any commercial or financial relationships that could be construed as a potential conflict of interest.
