# Supplementary material for: Magnetoencephalography for the Detection of Intervention Effects of a Specific Nutrient Combination in Patients with Mild Alzheimer’s Disease: Results from an Exploratory Double-Blind, Randomized, Controlled Study
Source: Front Neurol. 2016 Oct 17;7:161. doi: 10.3389/fneur.2016.00161 (PMC5065957; doi:10.3389/fneur.2016.00161)
Supplement: Supplementary file 1 [file Data_Sheet_1.DOCX]

Supplementary Material

**Magnetoencephalography for the detection of intervention effects of a specific nutrient combination in patients with mild Alzheimer’s disease: Results from an exploratory double blind randomised controlled study**

Elisabeth C.W. van Straaten^*^, Hanneke de Waal, Marieke M. Lansbergen, Philip Scheltens, Fernando Maestu, Rafal Nowak, Arjan Hillebrand, Cornelis J. Stam

*** Correspondence:** Elisabeth C.W. van Straaten i.vanstraaten@vumc.nl

# Supplementary Data

Appendix A. Descriptive statistics for occipital peak frequency; ITT MEG analysis population.

|  | ***Control***  *(n=27)* | ***Test***  *(n=22)* | ***P-value^1^*** |
| --- | --- | --- | --- |
| **Peak frequency (Hz)** |  |  |  |
| Baseline | 8.47 (1.46) [26] | 8.28 (1.37) [22] |  |
| Week 12 | 8.45 (1.08) [25] | 7.90 (1.51) [22] |  |
| Week 24 | 8.25 (1.25) [23] | 7.63 (1.42) [21] |  |
| 24-week trajectory |  |  | 0.115 |

*Note.* Data are means (SD) [N].

^1^ Mixed model for repeated measures (2 degrees of freedom contrast) with change-from-baseline value as an outcome, baseline as covariate.

Appendix B. Descriptive statistics for relative power; ITT MEG analysis population.

|  | | *Control (n = 27)* | | *Test (n = 22)* | | *P-value^1^* | |
| --- | --- | --- | --- | --- | --- | --- | --- |
| ***Delta [0.5-4 Hz]*** | | | | | | | |
| Baseline | | 0.281 (0.260, 0.302) (26) | | 0.278 (0.255, 0.301) (22) | |  | |
| Week 12 | | 0.282 (0.262, 0.302) (25) | | 0.286 (0.262, 0.311) (22) | |  | |
| Week 24 | | 0.284 (0.263, 0.304) (23) | | 0.293 (0.267, 0.319) (21) | |  | |
| 24-week trajectory | |  | |  | | 0.351 | |
| ***Theta [4-8 Hz]*** | | | | | | | |
| Baseline | | 0.194 (0.173, 0.214) (26) | | 0.201 (0.179, 0.222) (22) | |  | |
| Week 12 | | 0.198 (0.178, 0.218) (25) | | 0.202 (0.178, 0.225) (22) | |  | |
| Week 24 | | 0.199 (0.177, 0.221) (23) | | 0.203 (0.181, 0.225) (21) | |  | |
| 24-week trajectory | |  | |  | | 0.741 | |
| ***Alpha 1 [8-10 Hz]*** | | | | | | | |
| Baseline | | 0.102 (0.090, 0.113) (26) | | 0.098 (0.086, 0.110) (22) | |  | |
| Week 12 | | 0.099 (0.088, 0.110) (25) | | 0.097 (0.087, 0.108) (22) | |  | |
| Week 24 | | 0.098 (0.087, 0.109) (23) | | 0.098 (0.086, 0.111) (21) | |  | |
| 24-week trajectory | |  | |  | | 0.934 | |
| ***Alpha 2 [10-13 Hz]*** | | | | | | | |
| Baseline | | 0.098 (0.090, 0.107) (26) | | 0.097 (0.088, 0.106) (22) | |  | |
| Week 12 | | 0.096 (0.088, 0.104) (25) | | 0.096 (0.086, 0.106) (22) | |  | |
| Week 24 | | 0.097 (0.090, 0.105) (23) | | 0.093 (0.084, 0.103) (21) | |  | |
| 24-week trajectory | |  | |  | | 0.207 | |
| ***Beta [13-30 Hz]*** | | | | | | | |
| Baseline | | 0.255 (0.230, 0.279) (26) | | 0.257 (0.234, 0.280) (22) | |  | |
| Week 12 | | 0.253 (0.230, 0.275) (25) | | 0.251 (0.224, 0.278) (22) | |  | |
| Week 24 | | 0.252 (0.223, 0.280) (23) | | 0.245 (0.217, 0.272) (21) | |  | |
| 24-week trajectory | |  | |  | | 0.555 | |
| ***Gamma [30-48 Hz]*** | | | | | | | |
| Baseline | | 0.071 (0.062, 0.080) (26) | | 0.069 (0.062, 0.077) (22) | |  | |
| Week 12 | | 0.072 (0.063, 0.081) (25) | | 0.068 (0.061, 0.075) (22) | |  | |
| Week 24 | | 0.071 (0.061, 0.080) (23) | | 0.067 (0.060, 0.075) (21) | |  | |
| 24-week trajectory | |  | |  | | 0.625 | |

*Note.* Data are means (95% CI) (N).

^1^ Mixed model for repeated measures (2 degrees of freedom contrast) with change-from-baseline value as an outcome, baseline as covariate.

Appendix C. Descriptive statistics for mean phase lag index (PLI); ITT MEG analysis population.

|  | *Control (n = 27)* | *Test (n = 22)* | *P-value^1^* |
| --- | --- | --- | --- |
| ***Delta [0.5-4 Hz]*** | | | |
| Baseline | 0.110 (0.109, 0.112) (26) | 0.109 (0.107, 0.111) (22) |  |
| Week 12 | 0.108 (0.106, 0.111) (25) | 0.109 (0.107, 0.111) (22) |  |
| Week 24 | 0.109 (0.106, 0.111) (23) | 0.109 (0.107, 0.111) (21) |  |
| 24-week trajectory |  |  | 0.719 |
| ***Theta [4-8 Hz]*** | | | |
| Baseline | 0.094 (0.092, 0.096) (26) | 0.095 (0.093, 0.097) (22) |  |
| Week 12 | 0.095 (0.093, 0.097) (25) | 0.095 (0.093, 0.097) (22) |  |
| Week 24 | 0.095 (0.093, 0.097) (23) | 0.096 (0.094, 0.098) (21) |  |
| 24-week trajectory |  |  | 0.437 |
| ***Alpha 1 [8-10 Hz]*** | | | |
| Baseline | 0.135 (0.132, 0.138) (26) | 0.137 (0.135, 0.140) (22) |  |
| Week 12 | 0.135 (0.133, 0.138) (25) | 0.135 (0.133, 0.136) (22) |  |
| Week 24 | 0.135 (0.133, 0.137) (23) | 0.138 (0.136, 0.141) (21) |  |
| 24-week trajectory |  |  | 0.125 |
| ***Alpha 2 [10-13 Hz]*** | | | |
| Baseline | 0.110 (0.108, 0.112) (26) | 0.111 (0.110, 0.113) (22) |  |
| Week 12 | 0.110 (0.108, 0.111) (25) | 0.111 (0.109, 0.113) (22) |  |
| Week 24 | 0.110 (0.107, 0.113) (23) | 0.110 (0.108, 0.111) (21) |  |
| 24-week trajectory |  |  | 0.896 |
| ***Beta [13-30 Hz]*** | | | |
| Baseline | 0.049 (0.048, 0.051) (26) | 0.050 (0.048, 0.051) (22) |  |
| Week 12 | 0.049 (0.048, 0.050) (25) | 0.049 (0.048, 0.050) (22) |  |
| Week 24 | 0.050 (0.049, 0.051) (23) | 0.049 (0.048, 0.050) (21) |  |
| 24-week trajectory |  |  | 0.303 |
| ***Gamma [30-48 Hz]*** | | | |
| Baseline | 0.048 (0.047, 0.048) (26) | 0.048 (0.047, 0.048) (22) |  |
| Week 12 | 0.047 (0.047, 0.048) (25) | 0.048 (0.047, 0.048) (22) |  |
| Week 24 | 0.048 (0.047, 0.049) (23) | 0.048 (0.048, 0.049) (21) |  |
| 24-week trajectory |  |  | 0.976 |

*Note.* Data are means (95% CI) (N).

^1^ Mixed model for repeated measures (2 degrees of freedom contrast) with change-from-baseline value as an outcome, baseline as covariate.

Appendix D. Descriptive statistics for normalised clustering coefficient gamma; ITT MEG analysis population.

|  | *Control (n = 27)* | *Test (n = 22)* | *P-value^1^* |
| --- | --- | --- | --- |
| ***Delta [0.5-4 Hz]*** | | | |
| Baseline | 1.028 (1.025, 1.031) (26) | 1.026 (1.022, 1.031) (22) |  |
| Week 12 | 1.028 (1.024, 1.032) (25) | 1.026 (1.022, 1.029) (22) |  |
| Week 24 | 1.028 (1.024, 1.032) (23) | 1.027 (1.023, 1.031) (21) |  |
| 24-week trajectory |  |  | 0.879 |
| ***Theta [4-8 Hz]*** | | | |
| Baseline | 1.027 (1.023, 1.031) (26) | 1.025 (1.021, 1.029) (22) |  |
| Week 12 | 1.026 (1.021, 1.030) (25) | 1.030 (1.023, 1.036) (22) |  |
| Week 24 | 1.025 (1.022, 1.028) (23) | 1.028 (1.023, 1.032) (21) |  |
| 24-week trajectory |  |  | 0.128 |
| ***Alpha 1 [8-10 Hz]*** | | | |
| Baseline | 1.028 (1.024, 1.032) (26) | 1.029 (1.024, 1.034) (22) |  |
| Week 12 | 1.031 (1.027, 1.036) (25) | 1.031 (1.028, 1.034) (22) |  |
| Week 24 | 1.030 (1.026, 1.033) (23) | 1.030 (1.026, 1.034) (21) |  |
| 24-week trajectory |  |  | 0.944 |
| ***Alpha 2 [10-13 Hz]*** | | | |
| Baseline | 1.028 (1.023, 1.032) (26) | 1.024 (1.020, 1.029) (22) |  |
| Week 12 | 1.025 (1.021, 1.029) (25) | 1.029 (1.026, 1.031) (22) |  |
| Week 24 | 1.027 (1.023, 1.032) (23) | 1.026 (1.021, 1.031) (21) |  |
| 24-week trajectory |  |  | 0.028 |
| ***Beta [13-30 Hz]*** | | | |
| Baseline | 1.030 (1.027, 1.034) (26) | 1.029 (1.025, 1.033) (22) |  |
| Week 12 | 1.027 (1.024, 1.029) (25) | 1.028 (1.025, 1.031) (22) |  |
| Week 24 | 1.030 (1.026, 1.033) (23) | 1.027 (1.023, 1.032) (21) |  |
| 24-week trajectory |  |  | 0.237 |
| ***Gamma [30-48 Hz]*** | | | |
| Baseline | 1.023 (1.020, 1.025) (26) | 1.022 (1.018, 1.025) (22) |  |
| Week 12 | 1.024 (1.020, 1.028) (25) | 1.023 (1.020, 1.026) (22) |  |
| Week 24 | 1.024 (1.019, 1.028) (23) | 1.023 (1.019, 1.027) (21) |  |
| 24-week trajectory |  |  | 0.982 |

*Note.* Data are means (95% CI) (N).

^1^ Mixed model for repeated measures (2 degrees of freedom contrast) with change-from-baseline value as an outcome, baseline as covariate.

Appendix E. Descriptive statistics for normalised path length lambda; ITT MEG analysis population.

|  | *Control (n = 27)* | *Test (n = 22)* | *P-value^1^* |
| --- | --- | --- | --- |
| ***Delta [0.5-4 Hz]*** | | | |
| Baseline | 1.010 (1.007, 1.012) (26) | 1.008 (1.005, 1.012) (22) |  |
| Week 12 | 1.007 (1.004, 1.010) (25) | 1.009 (1.005, 1.013) (22) |  |
| Week 24 | 1.006 (1.003, 1.010) (23) | 1.008 (1.004, 1.012) (21) |  |
| 24-week trajectory |  |  | 0.387 |
| ***Theta [4-8 Hz]*** | | | |
| Baseline | 1.005 (1.002, 1.008) (26) | 1.008 (1.004, 1.011) (22) |  |
| Week 12 | 1.009 (1.006, 1.012) (25) | 1.011 (1.007, 1.015) (22) |  |
| Week 24 | 1.006 (1.003, 1.008) (23) | 1.011 (1.006, 1.016) (21) |  |
| 24-week trajectory |  |  | 0.167 |
| ***Alpha 1 [8-10 Hz]*** | | | |
| Baseline | 1.007 (1.005, 1.009) (26) | 1.010 (1.006, 1.014) (22) |  |
| Week 12 | 1.010 (1.007, 1.014) (25) | 1.010 (1.007, 1.012) (22) |  |
| Week 24 | 1.008 (1.005, 1.012) (23) | 1.012 (1.007, 1.017) (21) |  |
| 24-week trajectory |  |  | 0.372 |
| ***Alpha 2 [10-13 Hz]*** | | | |
| Baseline | 1.006 (1.002, 1.010) (26) | 1.007 (1.005, 1.010) (22) |  |
| Week 12 | 1.006 (1.003, 1.008) (25) | 1.009 (1.005, 1.013) (22) |  |
| Week 24 | 1.008 (1.003, 1.013) (23) | 1.007 (1.004, 1.011) (21) |  |
| 24-week trajectory |  |  | 0.382 |
| ***Beta [13-30 Hz]*** | | | |
| Baseline | 1.008 (1.005, 1.012) (26) | 1.009 (1.005, 1.013) (22) |  |
| Week 12 | 1.008 (1.005, 1.011) (25) | 1.008 (1.004, 1.012) (22) |  |
| Week 24 | 1.009 (1.006, 1.012) (23) | 1.007 (1.004, 1.010) (21) |  |
| 24-week trajectory |  |  | 0.371 |
| ***Gamma [30-48 Hz]*** | | | |
| Baseline | 1.004 (1.001, 1.007) (26) | 1.004 (1.001, 1.007) (22) |  |
| Week 12 | 1.004 (1.001, 1.007) (25) | 1.003 (1.000, 1.005) (22) |  |
| Week 24 | 1.004 (1.001, 1.008) (23) | 1.005 (1.001, 1.008) (21) |  |
| 24-week trajectory |  |  | 0.959 |

*Note.* Data are means (95% CI) (N).

^1^ Mixed model for repeated measures (2 degrees of freedom contrast) with change-from-baseline value as an outcome, baseline as covariate.

Appendix F. Descriptive statistics for MST normalised leaf number; ITT MEG analysis population.

|  | *Control (n = 27)* | *Test (n = 22)* | *P-value^1^* |
| --- | --- | --- | --- |
| ***Delta [0.5-4 Hz]*** | | | |
| Baseline | 0.535 (0.526, 0.544) (26) | 0.542 (0.531, 0.554) (22) |  |
| Week 12 | 0.542 (0.529, 0.554) (25) | 0.541 (0.534, 0.549) (22) |  |
| Week 24 | 0.548 (0.540, 0.555) (23) | 0.550 (0.538, 0.562) (21) |  |
| 24-week trajectory |  |  | 0.953 |
| ***Theta [4-8 Hz]*** | | | |
| Baseline | 0.542 (0.531, 0.554) (26) | 0.532 (0.520, 0.545) (22) |  |
| Week 12 | 0.536 (0.525, 0.547) (25) | 0.544 (0.532, 0.555) (22) |  |
| Week 24 | 0.538 (0.529, 0.547) (23) | 0.540 (0.529, 0.552) (21) |  |
| 24-week trajectory |  |  | 0.110 |
| ***Alpha 1 [8-10 Hz]*** | | | |
| Baseline | 0.551 (0.542, 0.560) (26) | 0.548 (0.536, 0.560) (22) |  |
| Week 12 | 0.543 (0.536, 0.551) (25) | 0.549 (0.537, 0.561) (22) |  |
| Week 24 | 0.545 (0.536, 0.554) (23) | 0.544 (0.531, 0.556) (21) |  |
| 24-week trajectory |  |  | 0.448 |
| ***Alpha 2 [10-13 Hz]*** | | | |
| Baseline | 0.539 (0.530, 0.549) (26) | 0.535 (0.524, 0.546) (22) |  |
| Week 12 | 0.541 (0.530, 0.551) (25) | 0.544 (0.533, 0.556) (22) |  |
| Week 24 | 0.542 (0.532, 0.552) (23) | 0.542 (0.532, 0.552) (21) |  |
| 24-week trajectory |  |  | 0.701 |
| ***Beta [13-30 Hz]*** | | | |
| Baseline | 0.543 (0.535, 0.551) (26) | 0.538 (0.529, 0.547) (22) |  |
| Week 12 | 0.540 (0.532, 0.549) (25) | 0.535 (0.524, 0.545) (22) |  |
| Week 24 | 0.534 (0.523, 0.545) (23) | 0.540 (0.529, 0.552) (21) |  |
| 24-week trajectory |  |  | 0.693 |
| ***Gamma [30-48 Hz]*** | | | |
| Baseline | 0.529 (0.522, 0.536) (26) | 0.533 (0.524, 0.541) (22) |  |
| Week 12 | 0.534 (0.525, 0.544) (25) | 0.532 (0.523, 0.541) (22) |  |
| Week 24 | 0.530 (0.520, 0.541) (23) | 0.535 (0.524, 0.546) (21) |  |
| 24-week trajectory |  |  | 0.723 |

*Note.* Data are means (95% CI) (N).

^1^ Mixed model for repeated measures (2 degrees of freedom contrast) with change-from-baseline value as an outcome, baseline as covariate.

Appendix G. Descriptive statistics for MST diameter; ITT MEG analysis population.

|  | *Control (n = 27)* | *Test (n = 22)* | *P-value^1^* |
| --- | --- | --- | --- |
| ***Delta [0.5-4 Hz]*** | | | |
| Baseline | 0.222 (0.216, 0.228) (26) | 0.220 (0.212, 0.228) (22) |  |
| Week 12 | 0.221 (0.215, 0.227) (25) | 0.224 (0.216, 0.233) (22) |  |
| Week 24 | 0.221 (0.214, 0.229) (23) | 0.217 (0.209, 0.224) (21) |  |
| 24-week trajectory |  |  | 0.842 |
| ***Theta [4-8 Hz]*** | | | |
| Baseline | 0.222 (0.216, 0.228) (26) | 0.225 (0.216, 0.233) (22) |  |
| Week 12 | 0.220 (0.211, 0.230) (25) | 0.220 (0.213, 0.228) (22) |  |
| Week 24 | 0.225 (0.219, 0.230) (23) | 0.221 (0.212, 0.231) (21) |  |
| 24-week trajectory |  |  | 0.778 |
| ***Alpha 1 [8-10 Hz]*** | | | |
| Baseline | 0.220 (0.212, 0.229) (26) | 0.221 (0.214, 0.229) (22) |  |
| Week 12 | 0.214 (0.208, 0.221) (25) | 0.218 (0.211, 0.225) (22) |  |
| Week 24 | 0.221 (0.216, 0.227) (23) | 0.219 (0.211, 0.227) (21) |  |
| 24-week trajectory |  |  | 0.732 |
| ***Alpha 2 [10-13 Hz]*** | | | |
| Baseline | 0.230 (0.221, 0.238) (26) | 0.219 (0.211, 0.227) (22) |  |
| Week 12 | 0.223 (0.216, 0.230) (25) | 0.222 (0.213, 0.231) (22) |  |
| Week 24 | 0.221 (0.214, 0.228) (23) | 0.219 (0.212, 0.227) (21) |  |
| 24-week trajectory |  |  | 0.992 |
| ***Beta [13-30 Hz]*** | | | |
| Baseline | 0.219 (0.213, 0.225) (26) | 0.222 (0.213, 0.231) (22) |  |
| Week 12 | 0.220 (0.214, 0.226) (25) | 0.219 (0.212, 0.226) (22) |  |
| Week 24 | 0.222 (0.213, 0.231) (23) | 0.221 (0.212, 0.230) (21) |  |
| 24-week trajectory |  |  | 0.951 |
| ***Gamma [30-48 Hz]*** | | | |
| Baseline | 0.227 (0.219, 0.234) (26) | 0.230 (0.222, 0.237) (22) |  |
| Week 12 | 0.222 (0.216, 0.228) (25) | 0.225 (0.218, 0.233) (22) |  |
| Week 24 | 0.226 (0.219, 0.234) (23) | 0.222 (0.214, 0.229) (21) |  |
| 24-week trajectory |  |  | 0.705 |

*Note.* Data are means (95% CI) (N).

^1^ Mixed model for repeated measures (2 degrees of freedom contrast) with change-from-baseline value as an outcome, baseline as covariate.

Appendix H. Descriptive statistics for MST tree hierarchy; ITT MEG analysis population.

|  | *Control (n = 27)* | *Test (n = 22)* | *P-value^1^* |
| --- | --- | --- | --- |
| ***Delta [0.5-4 Hz]*** | | | |
| Baseline | 0.399 (0.390, 0.409) (26) | 0.407 (0.397, 0.417) (22) |  |
| Week 12 | 0.406 (0.393, 0.418) (25) | 0.408 (0.400, 0.417) (22) |  |
| Week 24 | 0.412 (0.404, 0.420) (23) | 0.408 (0.400, 0.417) (21) |  |
| 24-week trajectory |  |  | 0.418 |
| ***Theta [4-8 Hz]*** | | | |
| Baseline | 0.401 (0.391, 0.412) (26) | 0.395 (0.382, 0.407) (22) |  |
| Week 12 | 0.397 (0.390, 0.405) (25) | 0.410 (0.403, 0.418) (22) |  |
| Week 24 | 0.400 (0.389, 0.410) (23) | 0.404 (0.394, 0.415) (21) |  |
| 24-week trajectory |  |  | 0.008 |
| ***Alpha 1 [8-10 Hz]*** | | | |
| Baseline | 0.410 (0.403, 0.417) (26) | 0.408 (0.398, 0.419) (22) |  |
| Week 12 | 0.404 (0.397, 0.410) (25) | 0.406 (0.395, 0.416) (22) |  |
| Week 24 | 0.402 (0.395, 0.410) (23) | 0.403 (0.393, 0.414) (21) |  |
| 24-week trajectory |  |  | 0.883 |
| ***Alpha 2 [10-13 Hz]*** | | | |
| Baseline | 0.402 (0.395, 0.409) (26) | 0.396 (0.388, 0.404) (22) |  |
| Week 12 | 0.402 (0.391, 0.412) (25) | 0.405 (0.392, 0.417) (22) |  |
| Week 24 | 0.406 (0.396, 0.415) (23) | 0.403 (0.391, 0.414) (21) |  |
| 24-week trajectory |  |  | 0.737 |
| ***Beta [13-30 Hz]*** | | | |
| Baseline | 0.409 (0.400, 0.418) (26) | 0.402 (0.391, 0.412) (22) |  |
| Week 12 | 0.407 (0.398, 0.416) (25) | 0.398 (0.387, 0.408) (22) |  |
| Week 24 | 0.397 (0.388, 0.406) (23) | 0.402 (0.392, 0.411) (21) |  |
| 24-week trajectory |  |  | 0.623 |
| ***Gamma [30-48 Hz]*** | | | |
| Baseline | 0.395 (0.385, 0.404) (26) | 0.406 (0.398, 0.414) (22) |  |
| Week 12 | 0.400 (0.394, 0.407) (25) | 0.401 (0.394, 0.409) (22) |  |
| Week 24 | 0.398 (0.390, 0.406) (23) | 0.403 (0.390, 0.416) (21) |  |
| 24-week trajectory |  |  | 0.906 |

*Note.* Data are means (95% CI) (N).

^1^ Mixed model for repeated measures (2 degrees of freedom contrast) with change-from-baseline value as an outcome, baseline as covariate.

Appendix I. Descriptive statistics for MST betweenness centrality; ITT MEG analysis population.

|  | *Control (n = 27)* | *Test (n = 22)* | *P-value^1^* |
| --- | --- | --- | --- |
| ***Delta [0.5-4 Hz]*** | | | |
| Baseline | 0.675 (0.666, 0.684) (26) | 0.670 (0.658, 0.683) (22) |  |
| Week 12 | 0.675 (0.664, 0.685) (25) | 0.666 (0.656, 0.676) (22) |  |
| Week 24 | 0.670 (0.658, 0.681) (23) | 0.678 (0.666, 0.689) (21) |  |
| 24-week trajectory |  |  | 0.227 |
| ***Theta [4-8 Hz]*** | | | |
| Baseline | 0.681 (0.670, 0.693) (26) | 0.679 (0.667, 0.691) (22) |  |
| Week 12 | 0.680 (0.669, 0.691) (25) | 0.666 (0.654, 0.679) (22) |  |
| Week 24 | 0.678 (0.666, 0.690) (23) | 0.672 (0.659, 0.685) (21) |  |
| 24-week trajectory |  |  | 0.219 |
| ***Alpha 1 [8-10 Hz]*** | | | |
| Baseline | 0.676 (0.665, 0.687) (26) | 0.676 (0.662, 0.689) (22) |  |
| Week 12 | 0.678 (0.668, 0.689) (25) | 0.683 (0.670, 0.696) (22) |  |
| Week 24 | 0.682 (0.671, 0.693) (23) | 0.678 (0.666, 0.690) (21) |  |
| 24-week trajectory |  |  | 0.571 |
| ***Alpha 2 [10-13 Hz]*** | | | |
| Baseline | 0.676 (0.667, 0.685) (26) | 0.681 (0.670, 0.692) (22) |  |
| Week 12 | 0.680 (0.667, 0.692) (25) | 0.678 (0.667, 0.689) (22) |  |
| Week 24 | 0.671 (0.660, 0.683) (23) | 0.678 (0.663, 0.693) (21) |  |
| 24-week trajectory |  |  | 0.943 |
| ***Beta [13-30 Hz]*** | | | |
| Baseline | 0.670 (0.657, 0.682) (26) | 0.675 (0.661, 0.688) (22) |  |
| Week 12 | 0.667 (0.658, 0.676) (25) | 0.678 (0.668, 0.688) (22) |  |
| Week 24 | 0.676 (0.665, 0.687) (23) | 0.677 (0.669, 0.685) (21) |  |
| 24-week trajectory |  |  | 0.206 |
| ***Gamma [30-48 Hz]*** | | | |
| Baseline | 0.675 (0.663, 0.688) (26) | 0.660 (0.648, 0.671) (22) |  |
| Week 12 | 0.670 (0.660, 0.681) (25) | 0.668 (0.655, 0.680) (22) |  |
| Week 24 | 0.672 (0.659, 0.684) (23) | 0.669 (0.655, 0.684) (21) |  |
| 24-week trajectory |  |  | 0.646 |

*Note.* Data are means (95% CI) (N).

^1^ Mixed model for repeated measures (2 degrees of freedom contrast) with change-from-baseline value as an outcome, baseline as covariate.

Appendix J. Descriptive statistics for MST eccentricity; ITT MEG analysis population.

|  | *Control (n = 27)* | *Test (n = 22)* | *P-value^1^* |
| --- | --- | --- | --- |
| ***Delta [0.5-4 Hz]*** | | | |
| Baseline | 0.171 (0.167, 0.176) (26) | 0.171 (0.165, 0.176) (22) |  |
| Week 12 | 0.170 (0.166, 0.175) (25) | 0.173 (0.167, 0.179) (22) |  |
| Week 24 | 0.171 (0.166, 0.177) (23) | 0.168 (0.162, 0.174) (21) |  |
| 24-week trajectory |  |  | 0.904 |
| ***Theta [4-8 Hz]*** | | | |
| Baseline | 0.172 (0.168, 0.176) (26) | 0.174 (0.167, 0.180) (22) |  |
| Week 12 | 0.171 (0.163, 0.178) (25) | 0.170 (0.165, 0.176) (22) |  |
| Week 24 | 0.173 (0.168, 0.177) (23) | 0.172 (0.164, 0.179) (21) |  |
| 24-week trajectory |  |  | 0.915 |
| ***Alpha 1 [8-10 Hz]*** | | | |
| Baseline | 0.171 (0.164, 0.177) (26) | 0.171 (0.165, 0.177) (22) |  |
| Week 12 | 0.167 (0.162, 0.171) (25) | 0.168 (0.163, 0.174) (22) |  |
| Week 24 | 0.171 (0.167, 0.175) (23) | 0.170 (0.163, 0.176) (21) |  |
| 24-week trajectory |  |  | 0.870 |
| ***Alpha 2 [10-13 Hz]*** | | | |
| Baseline | 0.177 (0.171, 0.183) (26) | 0.170 (0.163, 0.176) (22) |  |
| Week 12 | 0.172 (0.167, 0.177) (25) | 0.172 (0.165, 0.179) (22) |  |
| Week 24 | 0.171 (0.166, 0.177) (23) | 0.171 (0.165, 0.177) (21) |  |
| 24-week trajectory |  |  | 0.972 |
| ***Beta [13-30 Hz]*** | | | |
| Baseline | 0.169 (0.165, 0.174) (26) | 0.172 (0.165, 0.179) (22) |  |
| Week 12 | 0.170 (0.166, 0.175) (25) | 0.169 (0.164, 0.174) (22) |  |
| Week 24 | 0.171 (0.164, 0.178) (23) | 0.171 (0.164, 0.177) (21) |  |
| 24-week trajectory |  |  | 0.872 |
| ***Gamma [30-48 Hz]*** | | | |
| Baseline | 0.175 (0.169, 0.180) (26) | 0.177 (0.172, 0.182) (22) |  |
| Week 12 | 0.172 (0.167, 0.177) (25) | 0.175 (0.169, 0.181) (22) |  |
| Week 24 | 0.175 (0.170, 0.181) (23) | 0.172 (0.166, 0.177) (21) |  |
| 24-week trajectory |  |  | 0.629 |

*Note.* Data are means (95% CI) (N).

^1^ Mixed model for repeated measures (2 degrees of freedom contrast) with change-from-baseline value as an outcome, baseline as covariate.

Appendix K. Descriptive statistics for occipital peak frequency; N=6 subjects from Barcelona who had incompatible MEG data.

|  | *Control (n = 1)* | *Test (n = 5)* | |
| --- | --- | --- | --- |
| ***Peak frequency (in Hz)*** | | |  |
| Baseline | 10.44 (1.44) [1] | 7.93 (1.88) [5] | |
| Week 12 | 11.00 (1.05) [1] | 7.38 (1.44) [3] | |
| Week 24 | 8.71(1.44) [1] | 8.55 (1.38) [2] | |

*Note.* Data are means (SD) [N].

Appendix L. Descriptive statistics for relative power; N=6 subjects from Barcelona who had incompatible MEG data.

|  | *Control (n = 1)* | *Test (n = 5)* | |
| --- | --- | --- | --- |
| ***Delta [0.5-4 Hz]*** | | |  |
| Baseline | 0.20 (0.04) [1] | 0.22 (0.09) [5] | |
| Week 12 | 0.13 (0.03) [1] | 0.18 (0.08) [3] | |
| Week 24 | 0.30 (0.02) [1] | 0.21 (0.07) [2] | |
| ***Theta [4-8 Hz]*** | | |  |
| Baseline | 0.07 (0.01) [1] | 0.27 (0.13) [5] | |
| Week 12 | 0.17 (0.03) [1] | 0.31 (0.13) [3] | |
| Week 24 | 0.11 (0.03) [1] | 0.19 (0.05) [2] | |
| ***Alpha 1 [8-10 Hz]*** | | |  |
| Baseline | 0.03 (0.00) [1] | 0.12 (0.03) [5] | |
| Week 12 | 0.06 (0.02) [1] | 0.13 (0.03) [3] | |
| Week 24 | 0.05 (0.02) [1] | 0.13 (0.04) [2] | |
| ***Alpha 2 [10-13 Hz]*** | | |  |
| Baseline | 0.11 (0.02) [1] | 0.13 (0.05) [5] | |
| Week 12 | 0.23 (0.02) [1] | 0.14 (0.04) [3] | |
| Week 24 | 0.09 (0.02) [1] | 0.16 (0.04) [2] | |
| ***Beta [13-30 Hz]*** | | |  |
| Baseline | 0.52 (0.03) [1] | 0.21 (0.11) [5] | |
| Week 12 | 0.35 (0.05) [1] | 0.21 (0.05) [3] | |
| Week 24 | 0.39 (0.04) [1] | 0.25 (0.02) [2] | |
| ***Gamma [30-48 Hz]*** | | |  |
| Baseline | 0.06 (0.00) [1] | 0.04 (0.03) [5] | |
| Week 12 | 0.06 (0.01) [1] | 0.03 (0.01) [3] | |
| Week 24 | 0.07 (0.01) [1] | 0.06 (0.02) [2] | |

*Note.* Data are means (SD) [N].
